# Supplementary material for: Interprofessional Collaboration and Diabetes Management in Primary Care: A Systematic Review and Meta-Analysis of Patient-Reported Outcomes
Source: J Pers Med. 2022 Apr 15;12(4):643. doi: 10.3390/jpm12040643 (PMC9029958; doi:10.3390/jpm12040643)
Supplement: Supplementary file 1 [file jpm-12-00643-s001.zip › jpm-1660895-supplementary.pdf]

Interprofessional Collaboration and Diabetes Management in primary care: a Systematic review and Meta-analysis of Patient-Reported Outcomes

Search strategy

("Diabetes Mellitus, Type 2" OR “diabetes mellitus type 2” OR “DM 2” OR “DM II” OR “diabetes mellitus 2” OR “diabetes mellitus II”) AND

(((“Interprofession” OR “interprofessional” OR “inter-professional” OR “interdisciplinary” OR “interdisciplinar” OR “inter-disciplinary” OR “interinstitutional” OR “intersector” OR “inter sector” OR “intersectoral” OR “cross-sectoral” OR “interorganization” OR “interorganizational” OR “inter-organization” OR “inter-organizational” OR “interorganitation” OR “interorganitational” OR “multiprofessional” OR “multi-profession” OR “multi-professional” OR “multidiscipline” OR “multidisciplinary” OR “interpersonal” OR “transprofession” OR “transprofessional”)

AND (“collaboration” OR “collaborative” OR “practice” OR “care” OR “care service” OR “relation” OR “relations” OR “relationship” OR “team” OR “teams” OR “teamwork” OR “team care” OR “approach” OR “program” OR “programs” OR “intervention” OR “behavior” OR “communication” OR “cooperation” OR “participation” OR “work” OR “working” OR “management” OR “evaluation” OR “treatment”))

OR (“doctor nurse relationship” OR “practice nurse-GP team” OR “interprofessional collaboration” OR “IPC” OR “interdisciplinary collaboration” OR “intersectoral collaboration” OR “multiprofessional collaboration” OR “multidisciplinary collaboration” OR “transprofessional collaboration” OR “collaborative practice” OR “collaborative care” OR “ interprofessional team” OR “ interdisciplinary team” OR “multiprofessional team” OR “multidisciplinary team” OR “transprofessional team”)) AND

("patient reported outcome measures" OR “PROM” OR “PROMs” OR “PROs” OR “patient reported outcomes” OR “patient reported outcome” OR “patient reported outcome measure” OR “health related quality of life” OR “HRQOL” OR "quality of life")

Table S1. Summary characteristics of the included studies

Author, Year, Country	Source	Intervention team	Setting	Type of Interventions	Target of Intervention	Population	Follow-up	PROMs Analyzed	Results
Thankappan K.R. 2018, India	PLoS Medicine	- Nurse with a PhD in public health<br>- Medical Social Worker<br>- Trained lay people	Outpatient clinics	- Community engagement<br>- Peer support	Community-based	- 500 intervention group<br>- 507 control group	24 months	- SF-36 converted in Short Form 6 Dimension (SF-6D)	A community-based program didn't result in a nonsignificant reduction in diabetes incidence. However, there were significant improvements in some cardiovascular risk factors and physical functioning score of the HRQoL scale.
Penckofer S.M. 2012, USA	Annals of Behavioral Medicine	- Nurse<br>- Nurse trained by psychologist<br>- Psychologist	Outpatient clinics	- Psychological (psychoeducational)	Group-based	- 38 intervention group<br>- 36 control group	6 months	- SF-12<br>- CES-D<br>- QLI Diabetes version	SWEEP program provides evidence that group-therapy was more effective than usual care for treating depressed women with type 2 diabetes.
Chaveepojnkamjorn W. 2009, Thailand	Southeast Asian Journal of Tropical Medicine and Public Health	- Psychologist<br>- Diabetologist	Community Health Centers	- Educational (self-management)<br>- Empowerment-based<br>- Psychological (health belief model)<br>- Peer support	Group-based	- 80 intervention group<br>- 84 control group	6 months	- WHOQOL-BREF (THAI version)	This program, focused on enhancement of experience sharing among group members and participation in problem-solving, shows it is effective for improving perceived quality of life.
Piette J.D. 2011, USA	Medical Care	- Nurses with psychiatric and primary care training and experience, trained in CBT<br>- Experienced CBT supervisor and trainer	Primary Care Clinics	- Psychological (Cognitive-Behavioural Therapy)<br>- Telemedicine	Person-based	- 172 intervention group<br>- 177 control group	12 months	- SF-12<br>- BDI	This program of telephone delivered CBT combined with a pedometer-based walking program did not improve A1c values but significantly decreased patients' blood pressure, increased physical activity, and decreased depressive symptoms. The intervention also improved patients' functioning and quality of life.

Cezaretto A 2012, Brazil	Quality of Life Research	-Endocrinologist<br>- Psychologist<br>- Nutritionist<br>- Physical Educator	Primary Care Clinics	- Psychological (psychoeducational)	Person-based	- 97 intervention group<br>- 90 control group	9 months	- SF-36<br>- BDI	An intensive intervention on lifestyle with interdisciplinary approach for individuals at risk for type 2DM induced greater improvements in QoL than a traditional one, in parallel to better benefits on cardiometabolic profile.
Miklavcic J.J. 2020, Canada	BMC Geriatrics	- Nurse<br>- Dietitian<br>- Program Coordinator	Primary Care Clinics	- Educational (self-management)<br>- Community engagement	Community-based	- 70 intervention group<br>- 62 control group	6 months	- SF-12<br>- CES-D<br>- SEM-CD<br>- SDSCA	This pragmatic trial of a self-management intervention for older adults with T2DM and multimorbidity demonstrated inconclusive results for improving QoL.
Blackberry I.D. 2013, Australia	BMJ Online	- Practice nurses<br>- General Practitioner	General practices	- Psychological (coaching)<br>- Telemedicine<br>- Educational (self-management)	Person-based	- 30 intervention group<br>- 29 control group	18 months	- AQoL<br>- PHQ-9<br>- DSES	A telephone coaching by existing generalist practice nurses without prescribing rights found no evidence that was effective compared with usual primary care, either in reaching treatment targets or achieving more intensive treatment.
Du Pon E. 2019, Netherlands	BMC Endocrine Disorders	- Practice Nurses<br>- Dieticians specialized in diabetes care	General practices	- Educational (self-management)	Group-based	- 101 intervention group<br>- 100 control group	12 months	- EQ-5D-3L	PRISMA did not improve self-reported outcomes in patients with type 2 diabetes treated in primary care.It was not possible to make a statement about the clinical effects.
Vadstrup E.S. 2011, Denmark	Health Qual Life Outcomes	- Nurse<br>- Physiotherapist<br>- Podiatrist<br>- Dietician	Outpatient Clinics and General Practices	- Educational (self-management)<br>- Empowerment-based approach	Group-based	- 70 intervention group<br>- 73 control group	6 months	- SF-36	After 6 months this study suggests that a group-based rehabilitation programme is not superior to an individual counselling programme in changing patients' HRQOL and self-rated health.

Pauley T. 2016, Canada	Home Health Care Services Quarterly	- Nurse<br>- Personal Support Worker	Home	- Psychological (coaching)<br>- Educational (self-management)	Person-based	- 47 intervention group<br>- 47 control group	1 month	- DSES<br>- HADS	A PSW-led coaching intervention to improve diabetes self-efficacy shows no differences compared to nurse-led traditional standard of care. However, the results do demonstrate it may be sufficient to improve depression. Furthermore all subjects demonstrated significant improvements in self-efficacy measures.
Siaw M.Y.L. 2017, Singapore	Journal of Clinical Pharmacy and Therapeutics	- Physicians<br>- Diabetes Nurse educators<br>- Dietitians<br>- Clinical pharmacists	Outpatient clinics	- Medication control<br>- Educational (self-management)	Person-based	- 214 intervention group<br>- 197 control group	6 months	- DTSQ<br>- PAID	After 6 months a multidisciplinary collaborative care for Asian diabetic patients there was an increased QoL and satisfaction towards diabetes care, lightened the physicians' workload and cost saving. This study shows the effectiveness of this approach through an improvement of positive clinical, humanistic and economic outcomes.
Kulzer B. 2018, Germany	Diabetes Research and Clinical Practice	- Physicians received training based on a structured curriculum<br>- Medical staff	General practices	- Educational (self-management)	Person-based	- 440 intervention group<br>- 467 control group	12 months	- DTSQ	The iPDM process improved the use of diagnostic data leading to better glycemic control, more timely treatment adjustments (indicating reduced clinical inertia), and increased patient adherence and treatment satisfaction among patients and physicians.

Browning C. 2016, China	BMJ Open	- Community Doctors trained in coaching<br>- Community Nurses trained in coaching<br>- Community psychologists trained in coaching	Community health Centers	- Psychological (health coaching - motivational intervention)	Person-based	- 372 intervention group<br>- 339 control group	12 months	- SDSCA<br>- CDMSES	In this study, although a differential treatment effect was not observed for HbA1c, numerous outcomes (including HbA1c) improved in both groups, supporting the establishment of regular, free clinical health checks for people with T2DM in Chinese CHSs.
Markle-Reid M. 2017, Canada	Journal of the American Geriatrics Society	- Nurse<br>- Dietitian<br>- Program Coordinator (PC) from a community partner<br>- Peer volunteers	Primary Care Clinics	- Educational (self-management)<br>- Psychological (health coaching - motivational intervention)<br>- Individual and community engagement	Community-based and Group-based	- 80 intervention group<br>- 79 control group	6 months	- SF-12<br>- SDSCA<br>- CES-D<br>- SEM-CD	This study provide evidence that participation in a 6-month community-based intervention improved quality of life and self-management and reduced depressive symptoms in older adults with T2DM and comorbidity without increasing total healthcare costs.
van der Wulp I. 2012, Netherlands	Diabetic Medicine	- Expert patients trained in motivational interviewing<br>- General Practitioner<br>- Dieticians	General practices	- Peer support<br>- Psychological (self-management coaching)<br>- Patient engagement	Person-based	- 59 intervention group<br>- 60 control group	6 months	- CES-D<br>- DMSES<br>- PAID	A peer-led self-management coaching programme for recently diagnosed patients with Type 2 diabetes improved self-efficacy of patients experiencing low self-efficacy shortly after diagnosis
Davies M.J. 2008, UK	BMJ	- Dieticians<br>- Practice Nurses<br>- Nurse specialists	General practices	- Educational<br>- Patient empowerment	Group-based	- 437 intervention group<br>- 387 control group	12 months	- WHOQOL-BREF<br>- HADS<br>- PAID	A structured group education programme for patients with newly diagnosed type 2 diabetes resulted in greater improvements in weight loss and smoking cessation and positive improvements in beliefs about illness but no difference in haemoglobin

									A(1c) levels up to 12 months after diagnosis.
Cortez D.N. 2017, Brazil	BMC Public Health	- Lead researcher as a facilitator and an instigator of discussions<br>- Research assistant	Primary Care Clinics	- Educational<br>- Patient empowerment	Group-based	- 127 intervention group<br>- 111 control group	12 months	- SLC	The empowerment program based on individualized goals was effective in improving self-care practices and metabolic control of type 2 diabetes in Brazilian users
Kinmonth A.L. 1998, UK	BMJ	- Nurse<br>- General Practitioner	General practices	- Psychological	Person-based	- 142 intervention group<br>- 108 control group	12 months	- ADDQoL	A training programme in patient centred care for practitioners led to patients with newly diagnosed diabetes reporting better communication with doctors, greater wellbeing, and greater treatment satisfaction at one year, without loss of glycaemic control.
Lamers F. 2011, Netherlands	Journal of Advanced Nursing	- Nurses<br>- Psychiatrist<br>- General Practitioner<br>- Psychologist	General practices	- Psychological (Cognitive Behavioral Therapy)	Person-based	- 105 intervention group<br>- 103 control group	9 months	- DSC-R<br>- PAID	The nurse-administered intervention had limited effects on diabetes-specific quality of life.

Abbreviations: SF-36, short form health survey 36; SF-12, short form health survey 12; CES-D, The Center for Epidemiologic Studies Depression Tool; QLI Diabetes version, Diabetes version of the Ferrans and Powers Quality of Life Index; WHOQOL-BREF (THAI version), Thai abbreviated version of World Health Organization Quality of Life; BDI, Beck Depression Inventory; SEM-CD, Self-Efficacy for Managing Chronic Disease scale; SDSCA, Summary of Diabetes Self-Care Activities scale; AQoL, Assessment of Quality of Life Mark 2 instrument; PHQ-9, Major depressive syndrome; DMSES, 20-item Diabetes Management Self-Efficacy Scale; DSES, Diabetes Self-Efficacy Scale; EQ-5D-3L, EuroQol Five Dimensions scale, HADS, Hospital Anxiety and Depression Scale; DTSQ, Diabetes Satisfaction and Treatment Questionnaire; PAID, Problem Areas in Diabetes questionnaire; WHOQOL-BREF, short version of the World Health Organization Quality of Life instrument; SLC, Self-care for type 2 diabetes; ADDQoL, audit of diabetes dependent quality of life; DSC-R, DiabetesSymptom Checklist – Revised.

Table S2. Results of quality assessment process of Controlled Intervention studies.

**Author, Year, Country**	**1**	**2**	**3**	**4**	**5**	**6**	**7**	**8**	**9**	**10**	**11**	**12**	**13**	**14**	**Quality Rating**
Thankappan K.R. 2018, India	yes	yes	yes	yes	no	yes	yes	yes	yes	yes	yes	yes	yes	yes	good
Penckofer S.M. 2012, USA	yes	yes	yes	no	no	yes	yes	yes	yes	NR	yes	yes	yes	yes	good
Chaveepojnkamjorn W. 2009, Thailand	yes	yes	yes	NR	NR	yes	yes	yes	yes	NR	yes	yes	yes	NR	fair
Piette J.D. 2011, USA	yes	yes	yes	NR	NR	yes	yes	yes	yes	NR	yes	yes	yes	yes	good
Cezaretto A 2012, Brazil	yes	yes	NR	NR	NR	yes	no	yes	yes	NR	yes	no	yes	NR	poor
Markle-Reid M. 2017, Canada	yes	yes	yes	no	yes	yes	yes	yes	yes	NR	yes	yes	yes	yes	good
Miklavcic J.J. 2020, Canada	yes	yes	yes	no	yes	no	yes	yes	yes	NR	yes	yes	yes	yes	good
Blackberry I.D. 2013, Australia	yes	yes	yes	no	yes	yes	yes	yes	yes	NR	yes	yes	yes	NR	good
van der Wulp I. 2012, Netherlands	yes	yes	yes	NR	NR	yes	yes	yes	yes	NR	yes	yes	yes	NR	fair
Davies M.J. 2008, UK	yes	yes	yes	NR	NR	yes	no	yes	yes	NR	yes	yes	yes	yes	fair
Du Pon E. 2019, Netherlands	yes	yes	yes	NR	NR	yes	yes	yes	yes	NR	yes	yes	yes	yes	good
Cortez D.N. 2017, Brazil	yes	yes	yes	NR	NR	yes	yes	yes	yes	NR	yes	yes	yes	NR	fair
Vadstrup E.S. 2011, Denmark	yes	yes	yes	no	yes	yes	yes	yes	yes	NR	yes	yes	yes	yes	good
Pauley T. 2016, Canada	yes	yes	yes	no	yes	yes	yes	yes	yes	NR	yes	NR	yes	yes	good
Siaw M.Y.L. 2017, Singapore	yes	yes	yes	NR	NR	yes	yes	no	no	NR	yes	yes	yes	yes	fair
Kulzer B. 2018, Germany	yes	yes	yes	NR	NR	yes	yes	yes	yes	NR	yes	yes	yes	yes	good
Browning C. 2016, China	yes	yes	yes	no	yes	yes	yes	yes	yes	NR	yes	yes	yes	NR	good
Kinmonth A.L. 1998, UK	yes	yes	yes	no	yes	yes	no	yes	yes	NR	yes	yes	yes	yes	good
Lamers F. 2011, Netherlands	yes	yes	yes	NR	yes	yes	yes	no	yes	NR	yes	yes	yes	yes	good

Abbreviation: NR, not reported. Author-derived key for standardization: ≤ 5 POOR, 6-7 FAIR, ≥ 8 GOOD.
